# Addressing fatty tissue in quantitative susceptibility mapping of human knee cartilage

**DOI:** 10.1007/s10334-025-01280-0

**Published:** 2025-07-24

**Authors:** Cornelia Säll, Emelie Lind, Emma Einarsson, Aleksandra Turkiewicz, Martin Englund, Pernilla Peterson

**Affiliations:** 1 Imaging and Physiology, Skåne University Hospital, Lund, Sweden; 2 Department of Medical Radiation Physics, Lund University, Lund, Sweden; 3 Medical Radiation Physics, Department of Translational Medicine, Lund University, Malmö, Sweden; 4 Clinical Epidemiology Unit, Orthopedics, Department of Clinical Sciences Lund, Lund University, Lund, Sweden

**Keywords:** QSM, Cartilage, Osteoarthritis, Fatty tissue

## Abstract

**Objective:**

To evaluate the effects of excluding fatty tissue in QSM of human knee cartilage.

**Materials and methods:**

Gradient echo images from 18 knee-healthy volunteers were acquired, from which chemical shift corrected field perturbation maps were calculated. Based on these, QSM maps were reconstructed using morphology enabled dipole inversion and one of three masking alternatives: (1) excluding no tissue, (2) excluding bone marrow, and (3) excluding all fatty tissues. The slope of a linear regression [ppm/%] between susceptibility values and the relative distance from the bone surfaces was used as a measurement of contrast between cartilage layers. The average differences in slopes between methods are reported with 95% confidence intervals.

**Results:**

The expected susceptibility differences between cartilage layers from literature were observed for all tested reconstruction techniques. However, smaller slopes (average difference (confidence interval)) were detected when either all fatty tissue (− 0.090 (− 0.121, − 0.059) ppm/%) or bone marrow (− 0.088 (− 0.121, − 0.055) ppm/%) was excluded from reconstruction.

**Discussion:**

All tested methods result in adequate image quality in QSM of knee cartilage. However, exclusion of fatty tissue decreased the susceptibility contrast between cartilage layers. Assuming that phase contributions from chemical shift are addressed, inclusion of fatty tissue may be preferable.

## Introduction

Osteoarthritis (OA) is a common degenerative joint disorder, most often affecting the knee [1, 2]. The existing treatment alternatives are very limited [3] and better understanding of the early processes of the disease is needed to develop new diagnostic and therapeutic strategies. To meet this need, several non-invasive MRI-based approaches to assess cartilage quality have been used to probe proteoglycans, water, and collagen, including dGEMRIC, gagCEST, and T2-mapping [4].

**Fig. 1:**
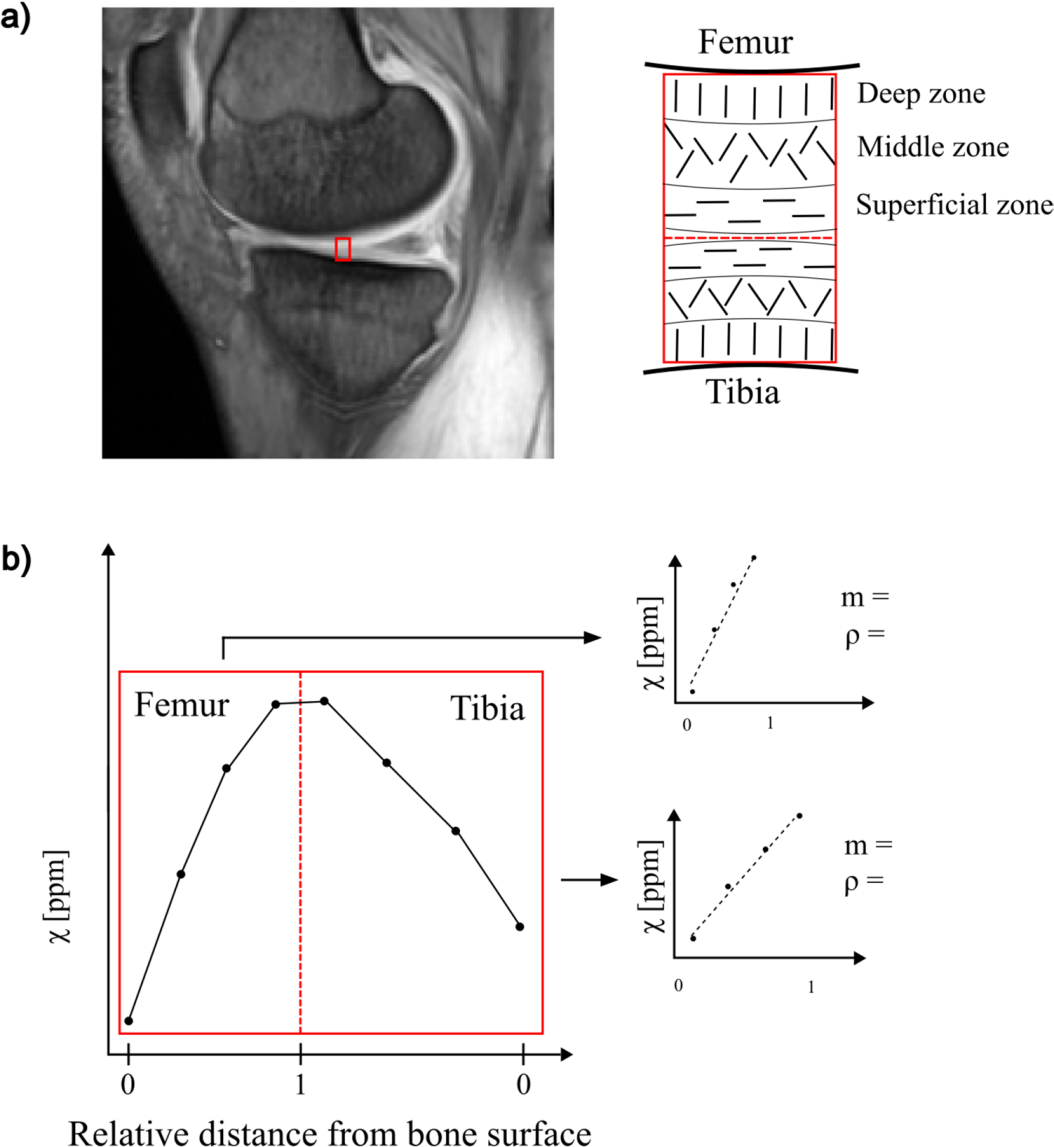
**a** Overview of the articular cartilage analyzed. Left: morphological image showing the medial condyle, and the articular cartilage. The red square represents the ROI. Right: a schematic drawing of the articular cartilage, with the collagen fiber orientation in each of the three layers shown. This structure has been suggested to affect the QSM contrast. **b** Visualization of data extraction and analysis. Left: a profile of susceptibility values versus relative distance from the femoral and tibial bone surfaces extracted from the ROI. Each point corresponds to the mean of susceptibility values in the rows of the ROI. Right: data analysis was performed on the femoral and tibial cartilage separately. Susceptibility values were plotted against the distance from each bone surface. From each cartilage, a slope (m) and correlation coefficient (ρ) were extracted

In addition, quantitative susceptibility mapping (QSM) [5–13] has lately been suggested as a method to obtain information about the cartilage collagen structure. Healthy articular cartilage has a highly ordered collagen structure with three layers, the deep, middle, and superficial zone, each characterized by the orientation of its collagen fibers [14, 15]; see Fig. 1a. Earlier studies have suggested that QSM may reveal this multilayered structure of the articular cartilage [6], and that degeneration of the articular cartilage in OA appears to alter the QSM contrast between layers as a result of changes in the collagen matrix structure [7, 12].

In QSM, the signal phase data are used to estimate magnetic susceptibility values. However, the signal phase is also affected by the chemical shift and the multiple frequency components of the signal from fat [16]. This issue has typically been addressed by excluding fatty tissue from the reconstruction process by application of a mask, especially in imaging of the brain [17]. In body imaging, where more fatty tissue is present, it has instead been suggested to account for the phase effects of the fatty signal components using chemical-shift encoded imaging (CSEI) [18–20].

Addressing the impact of chemical shift may be of importance also for application of QSM in knee cartilage due to the proximity of several fatty tissue depots, such as bone marrow adipose tissue, Hoffa’s fat pad, and subcutaneous adipose tissue. Yet, fatty tissue has most often been excluded in the QSM reconstruction [7, 8, 10, 13]. This choice has several disadvantages. First, masking to exclude specific tissue may be impractical in larger studies as individualized and precise definition of the mask is needed to ensure minimal cartilage volume loss. Second, masking may affect the accuracy of the technique as the susceptibility of the excluded tissue may not be fully accounted for in the following reconstruction. It may also make background field removal challenging as cartilage is in the immediate vicinity of the edges of the mask [21]. As an alternative to exclusion of fatty tissue, a few studies have corrected the impact of chemical shift using CSEI [9], or using a combination of masking and CSEI [6]. However, the effects of masking have not been evaluated, nor have the various approaches of handling fatty tissue in QSM of knee cartilage been compared.

Focusing on the articular cartilage of the knee, the purpose of this work is to evaluate the effects of excluding fatty tissue from the QSM reconstruction. Three masking alternatives are evaluated using in vivo data acquired from knee-healthy subjects at 7 T. Additionally, three background field removal techniques are compared.

## Methods

In this study, the effects of excluding fatty tissue in QSM reconstruction of the knee were evaluated from in vivo data. Three masking alternatives were compared: (1) excluding no tissue, (2) excluding bone marrow, and (3) excluding all fatty tissues. For all masking options, the chemical shifts of multiple fat frequency components were addressed using CSEI. QSM reconstruction was made using morphology enabled dipole inversion (MEDI) [22–25], comparing three background field removal techniques: variable-kernel sophisticated harmonic artifact reduction for phase data (V-SHARP) [26], projection onto dipole fields (PDF) [27], and Laplacian boundary value (LBV) [28]. In addition, results in the medial and lateral tibiofemoral compartments were compared.

**Fig. 2:**
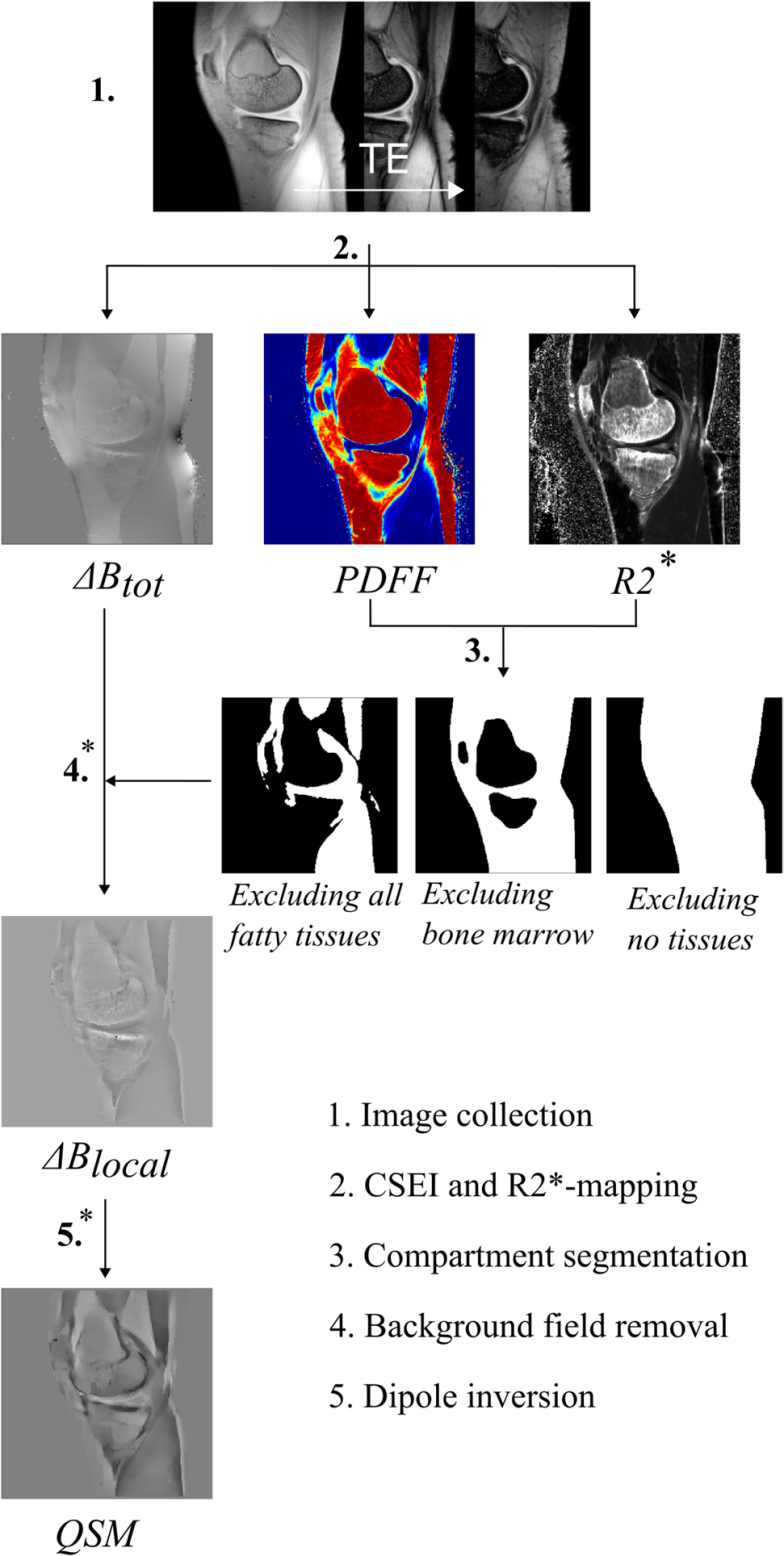
A flowchart describing the steps performed in the acquisition and post processing of data for QSM reconstruction. The processing steps where masks were applied are indicated by *

All data analysis and post processing were performed using MATLAB [The MathWorks Inc. (2023). MATLAB version: 9.8 (R2020a) Natick, MA USA]. The data acquisition and analysis are summarized in Fig. 2.

### Human subjects

Image data from 23 participants without known OA were collected with permission from the ethics review authority and after written informed consent. For each participant, the knee to be imaged was selected randomly. Before image acquisition, a Knee Osteoarthritis Outcome Score (KOOS) questionnaire was filled out by each participant. Based on the KOOS scores, asymptomatic subjects were identified as described in Ref [29]. Briefly, 42 questions separated into five categories (quality-of-life, pain, symptoms, activities of daily living and sport and recreation function) were answered using a five-grade scale. The answers were converted to five scores between 0 and 100, where a lower score indicated more severe OA symptoms. Scores below a defined cutoff for quality of life and any two of the other categories were taken as an indication of a symptomatic knee, see cut-off values for each category in ref [29]. In this analysis, which was part of a larger study, only data from asymptomatic participants were included. Further, one subject was excluded due to the presence of red bone marrow resulting in a low fat fraction of the femoral bone marrow and four subjects were excluded due to fat/water swaps in the CSEI reconstruction. This yielded data from a total of 18 participants, 6 men and 12 women, aged between 34 and 61 years (mean 47.3 years).

### Image acquisition

Image acquisition was performed on an actively shielded 7 T Philips MR system (Achieva, Philips Healthcare, Best, NL), using a QED Knee Coil 1TX/28RX (Quality Electrodynamics LCC, Mayfield Village, OH USA), step 1 in Fig. 2. Two sagittal and interleaved multi-echo gradient echo sequences, each with bipolar read-out gradients and eight echo times, such that TE_1, sequence 1_/ΔTE = 1.2/1.2 ms and TE/ΔTE = 1.8/1.2 ms, were collected and combined, yielding ΔTE_effective_ = 0.6 ms. The following parameters were used: TR = 30 ms, matrix size = 256 × 256 × 37, voxel size = 0.58 × 0.58 × 3 mm^3^, flip angle = 8°, SENSE acceleration factor = 1.2, and bandwidth = 1378 Hz/pixel. The phase and magnitude of the signal was obtained along with a B0 map from the mDixon Quant application (Philips Healthcare, Best, NL).

### Data processing and reconstruction of susceptibility maps

#### Chemical shift encoded imaging

Based on the collected data, a proton density fat fraction map (PDFF), and a chemical-shift corrected total field perturbation map (Δ*B*_tot_), was calculated for each participant using CSEI (step 2 in Fig. 2). The iterative linear least-squares reconstruction included an a priori fat model with *M* = 8 peaks with relative amplitudes ${\alpha }_{m}$ and frequency differences compared to water $\Delta {f}_{m}$, as well as estimation of R2* and phase errors due to the interleaved ${\theta }_{\text{int}}$ and bipolar ${\theta }_{\text{bip}}$ acquisition [30, 31], using the following signal equation:

1 $$\begin{array}{c}S\left(TE,n,p\right)=\left(W+F\sum_{m=1}^{M}{\alpha }_{m}{e}^{i2\pi\Delta {f}_{m}TE}\right){e}^{i2\pi \psi TE}{e}^{{\left(-1\right)}^{n}i\pi {\theta }_{bip}}{e}^{pi\pi {\theta }_{\text{int}}},\end{array}$$

where *W* and *F* denote water and fat, *n* the number of the echo in each echo train, and *p* = − 1 for the first interleave and *p* = 1 for the second interleave. Here, the $\Delta {B}_{\text{tot}}$ and R2* are obtained from the real and imaginary part of the complex field map $\psi$, according to [32]

2 $$\begin{array}{c}\psi =\Delta {B}_{\text{tot}}+\frac{iR{2}^{*}}{2\pi }.\end{array}$$

The complex field map is constructed, such that

3 $$\begin{array}{c}{e}^{i2\pi \psi TE}={e}^{i2\pi \left(\Delta {B}_{tot}+\frac{iR{2}^{*}}{2\pi }\right)TE}={e}^{i2\pi\Delta {B}_{tot}}{e}^{-R{2}^{*}TE}.\end{array}$$

The mDixon B0 map, in some cases slightly shifted in frequency to avoid fat–water swaps, was used as an initial guess. Also, an R2*-map was calculated using the ARLO function [33] from the MEDI toolbox [22–25].

#### Segmentation and mask definition

For each participant, three masks were defined (step 3 in Fig. 2):

1. *excluding no tissue:* obtained by thresholding based on the magnitude image collected with TE = 1.2 ms.
2. *excluding bone marrow:* obtained by defining bone marrow regions and excluding them from the first mask. This was done by first defining all fatty tissues using a threshold level of PDFF > 70%. From this, bone marrow in femur, tibia, fibula, and patella was separated from other fatty tissues using region growing in the R2*-map.
3. *excluding all fatty tissues:* obtained by thresholding voxels with a fat fraction larger than 35% in the PDFF map, and excluded from the first map.

Threshold values in the magnitude and PDFF images were determined based on visual inspection of images. In some cases, manual corrections of masks were needed to ensure satisfactory definition of the tissues. Extra attention was given to make sure that: (1) air was not included in the mask, (2) a good correspondence was obtained between the marrow regions of the mask and the marrow regions of the PDFF maps, and (3) the articular cartilage between femur and tibia was not lost.

#### QSM reconstruction

The chemical-shift corrected total field perturbation map Δ*B*_tot_ from CSEI was used to calculate the magnetic susceptibility using the MEDI toolbox. First, background field removal was performed with each of the three techniques, V-SHARP, PDF, and LBV, yielding the local field perturbation (Δ*B*_local_), see step 4 in Fig. 2, where V-SHARP was used via SEPIA [34]. For all, standard settings were used with the following exceptions: (1) For PDF, the maximum number of iterations was set to 100, and (2) for V-SHARP, a minimum radius of 1 mm and a maximum radius of 10 mm were used.

Second, dipole inversion was performed using MEDI with a regularization parameter *λ* = 200; see step 5 in Fig. 2.

Erosion of the mask was performed throughout the reconstruction steps, to minimize artifacts stemming from the edge regions [35]. Around the air–tissue interface, an erosion of three pixels was performed before background field removal, and an erosion of two pixels before the dipole inversion step. At the fatty–lean tissue interface, however, smaller erosion was necessary to avoid loss of the articular cartilage volume. Here, erosion of only one pixel was performed before each of the background field removal and dipole inversion steps.

### Data analysis

In each susceptibility map, rectangular regions of interests (ROIs) with an anterior–posterior width of five pixels were defined in one medial and one lateral slice, respectively, covering the articular cartilage between the femoral and tibial bone surfaces. In each ROI, the susceptibility values were shifted by addition/subtraction, so that the value at the cartilage surface was set to zero to make it possible to visually compare the relative susceptibility values obtained from QSM. Each ROI was manually divided into a femoral and tibial part based on magnitude data.

Susceptibility profiles were visualized by plotting susceptibility values within ROIs against the relative distance from the bone surfaces, see Fig. 1b. As no ground truth was known, and could thus not be used for comparison, the results were evaluated based on two assumptions: (1) that susceptibility should increase with distance from the bone surfaces [6, 7] and (2) that the profile over the cartilage should be constant within subject and compartment, and thus not affected by the choice of masking method. The quality of the susceptibility profile for each participant and for each of the femoral and tibial compartments was thus evaluated using two measures; see Fig. 1b.

First, *the slope of a linear regression* between susceptibility and relative distance from the bone surface was used as a measure of contrast between cartilage layers [ppm/%]. A positive slope indicates the expected increase in susceptibility values going from the bone to the cartilage surface. Assuming a linear relationship between susceptibility and relative distance from the bone surface is a simplified model of the complex cartilage structure but is motivated by the low imaging resolution and thus few data points available in each compartment.

Second, *Pearson’s correlation coefficient* was estimated and used in two ways. First, as an indication of whether a linear model is a reasonable description of the association between susceptibility and distance from the bone surface in vivo, and second, to evaluate to what extent unexpected susceptibility profiles were present, which may possibly be related to artifacts in the QSM images.

The methods were compared using a Bland–Altman analysis, using the masking alternative of excluding no tissue with V-SHARP background field removal as a reference. For each comparison, the average difference and upper and lower limit of agreement (LoA) were estimated with 95% confidence interval (CI).

## Results

### Comparing masking alternatives

**Fig. 3:**
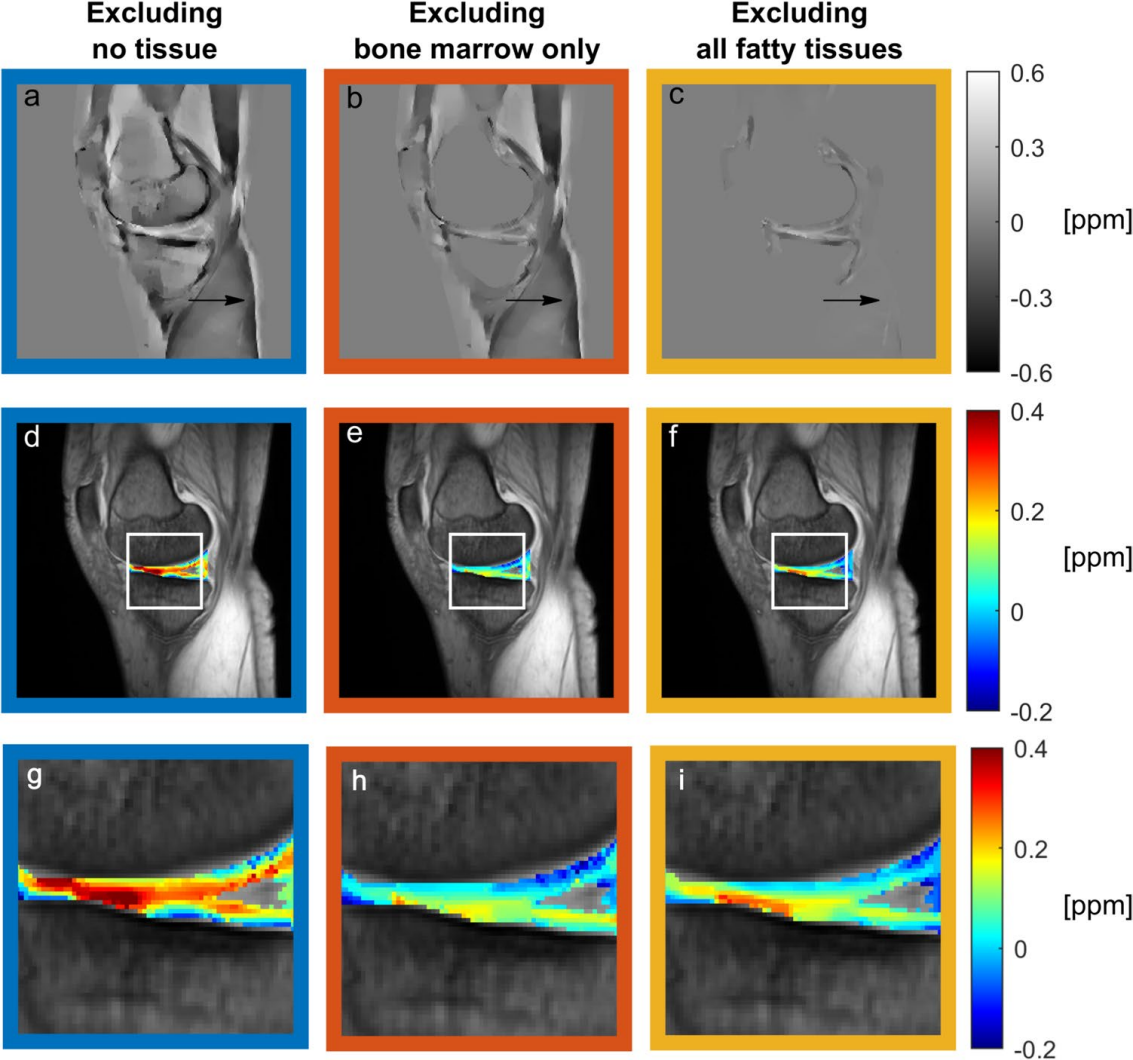
**a**–**c** Susceptibility maps obtained for one knee, **d**–**f** susceptibility values in the cartilage area overlaid on the magnitude image for reference, and **g**–**i** the cartilage area magnified. All three masking alternatives are shown: **a, d, g** excluding no tissue, **b, e, h** excluding bone marrow only, and **c, f, i** excluding all fatty tissues. Arrows point out an area with observed shading artifacts

Overall, image quality was adequate; see Fig. 3. When fatty tissues were not excluded from the reconstruction process, shading artifacts were visible; see arrows in Fig. 3a, b. These were mitigated by exclusion of all fatty tissues; see arrow in Fig. 3c. Visually, a gradient of susceptibility values over the cartilage area was seen. This was especially prominent when no tissue was excluded from the reconstruction.

**Fig. 4:**
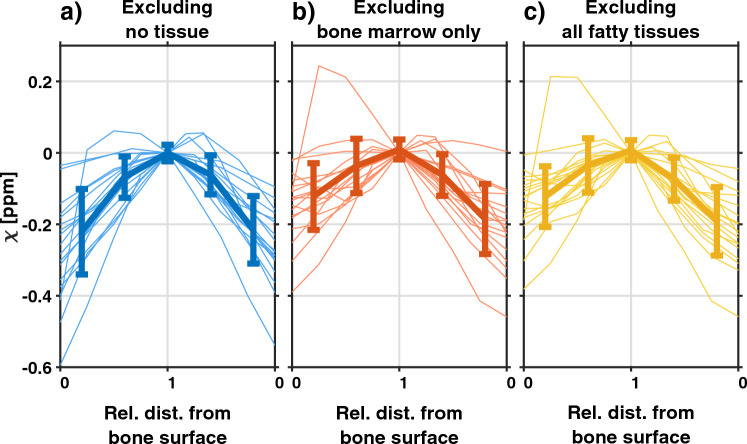
Susceptibility values versus relative distance from the bone surface extracted from the medial femoral and tibial cartilage when excluding **a** no tissue, **b** bone marrow, and **c** all fatty tissues. The profiles of each participant are shown as thin lines, and the mean susceptibility values of all participants at five segments of the full cartilage depth are shown as a bold line, with the standard deviation indicated by the error bars. The values at the cartilage surface were shifted to zero ppm

To further evaluate the contrast between cartilage layers, the profiles of susceptibility values obtained from the ROIs using V-SHARP are shown in Fig. 4. For all masking alternatives, the expected increase of susceptibility with distance from the bone surface was seen, but with a large inter-subject variability. The average (± standard deviation) slope for each masking alternative was 0.27 ppm/% ± 0.12 ppm/% when excluding no tissue, 0.18 ppm/% ± 0.10 ppm/% when excluding bone marrow, and 0.18 ppm/% ± 0.09 ppm/% when excluding all fatty tissues.

**Fig. 5:**
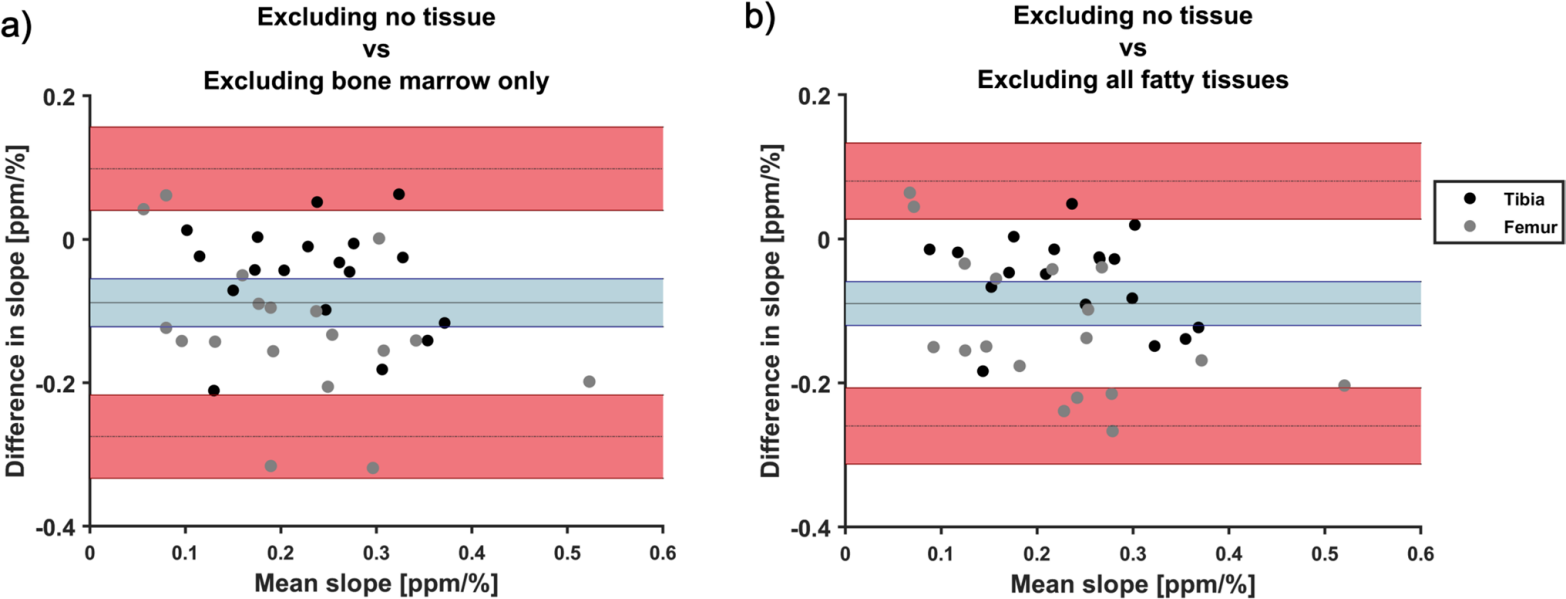
Bland–Altman plots showing the mean differences between slopes (tested method−reference) as a function of mean slope between methods. Comparison was made between excluding no tissue with V-SHARP and **a** excluding bone marrow and **b** excluding all fatty tissues. Mean difference with 95% confidence interval is given by the solid line and blue area, and limits of agreement with 95% confidence intervals are given by dashed lines and red areas. Data points from the femoral cartilage are shown in black, and data points from the tibial cartilage are shown in gray. In both cases, the analysis shows that the masking techniques are not interchangeable

Comparing the slopes between masking alternatives using Bland–Altman analysis, we found that the masking alternatives were not interchangeable with a −0.1 ppm/% smaller slope when either bone marrow or all fatty tissues were excluded, compared to when no tissue was excluded (see Fig. 5 and Table 1). The LoA were large, and comparable to the size of the slopes. Results using the two masking alternatives where fatty tissues were excluded from reconstruction were similar.

**Table 1:** Mean difference in slope relative to the reference technique with upper and lower limits of agreement with 95% confidence interval obtained medially and laterally using the tested masking alternatives and background field removal techniques in vivo

	Background field removal	Masking alternative compared to reference (excluding no tissue + V-SHARP)	Mean difference (95% CI)	Lower limit of agreement (95% CI)	Upper limit of Agreement (95% CI)
Medial	V-SHARP	Excluding no tissue	Reference	Reference	Reference
		Excluding bone marrow	− 0.088 (− 0.121, − 0.055)	− 0.275 (− 0.333, − 0.217)	0.099 (0.041, 0.157)
		Excluding all fatty tissues	− 0.090 (− 0.121, − 0.059)	− 0.260 (−0.207, −0.313)	0.080 (0.027, 0.133)
	PDF	Excluding no tissue	0.003 (− 0.009, 0.015)	− 0.065 (− 0.086, − 0.044)	0.071 (0.050, 0.092)
		Excluding bone marrow	− 0.053 (− 0.098, − 0.008)	− 0.306 (− 0.384, − 0.228)	0.199 (0.121, 0.278)
		Excluding all fatty tissues	− 0.054 (− 0.083, − 0.025)	− 0.215 (− 0.266, − 0.165)	0.108 (0.058, 0.158)
	LBV	Excluding no tissue	0.002 (− 0.011, 0.014)	−0.066 (− 0.087, − 0.045)	0.069 (0.048, 0.090)
		Excluding bone marrow	− 0.002 (− 0.045, 0.041)	− 0.243 (− 0.318, − 0.168)	0.239 (0.164, 0.314)
		Excluding all fatty tissues	− 0.007 (− 0.042, 0.028)	− 0.186 (− 0.246, − 0.126)	0.199 (0.139, 0.259)
Lateral	V-SHARP	Excluding no tissue	Reference	Reference	Reference
		Excluding bone marrow	− 0.062 (− 0.084, − 0.040)	− 0.197 (− 0.225, − 0.148)	0.062 (0.023, 0.101)
		Excluding all fatty tissues	− 0.053 (− 0.071, − 0.035)	− 0.154 (− 0.185, − 0.122)	0.049 (0.017, 0.081)
	PDF	Excluding no tissue	− 0.017 (− 0.028, − 0.006)	− 0.081 (− 0.101, − 0.061)	0.047 (0.027, 0.067)
		Excluding bone marrow	− 0.008 (− 0.034, 0.018)	− 0.152 (− 0.198, 0.108)	0.137 (0.092, 0.183)
		Excluding all fatty tissues	− 0.018 (− 0.042, 0.006)	− 0.151 (− 0.192, − 0.110)	0.114 (0.073, 0.155)
	LBV	Excluding no tissue	− 0.009 (− 0.020, 0.002)	− 0.072 (− 0.092, − 0.052)	0.053 (0.033, 0.073)
		Excluding bone marrow	− 0.002 (− 0.027, 0.023)	− 0.141 (−0.183, −0.097)	0.136 (0.087, 0.173)
		Excluding all fatty tissues	− 0.001 (− 0.021, 0.019)	− 0.110 (− 0.143, − 0.076)	0.108 (0.074, 0.142)

**Fig. 6:**
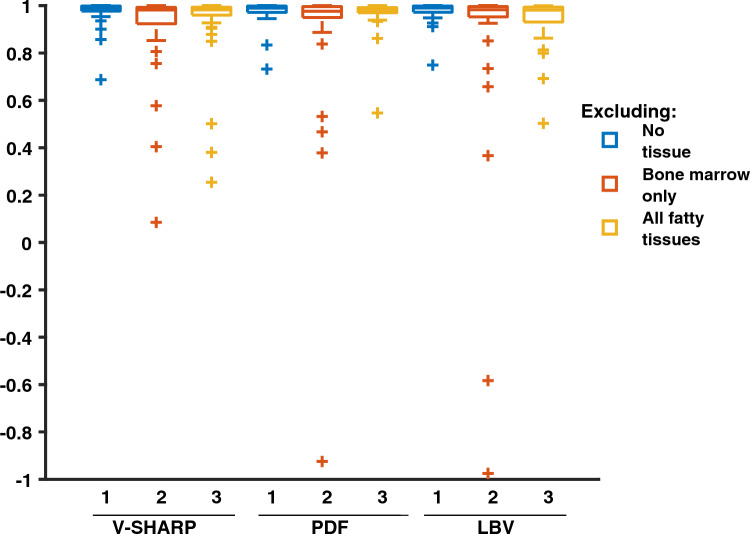
Boxplot of the correlation coefficients between susceptibility and relative distance to the bone surface when excluding no tissue (blue), excluding bone marrow only (red) and excluding no fatty tissues (yellow), for the three background field removal techniques as indicated in the plot. An accurate technique was expected to yield high and positive correlation coefficients

The correlation coefficients between susceptibility and the relative distance from the bone surface were generally high (see Fig. 6), indicating that the linear fit is a reasonable model for susceptibility values across cartilage depth in this data set. Overall, a larger span of correlation coefficients was seen when fatty tissues were excluded compared to when fatty tissues were included, possibly indicating a higher level of noise or artifacts. Cases of low correlation coefficients were especially present when bone marrow only was excluded from reconstruction.

### Comparing background field removal techniques

When fatty tissue was included in the reconstruction, no systematic differences in slopes were seen between background field removal techniques. Further, the comparison of background field removal techniques yielded narrower LoA than the comparison between different masking alternatives (see Table 1). Thus, the choice of masking alternative may have a larger effect on the results than the choice of background field removal technique.

When comparing masking alternatives in combination with other background field removal techniques, slight differences in the results were seen. Similar to the results obtained using V-SHARP, lower slopes were seen when bone marrow or all fatty tissues were excluded combined with PDF (see Table 1). This, however, was not observed for LBV, where similar slopes were obtained for all three masking techniques (see Table 1).

The correlation coefficients obtained following the use of PDF and LBV were also high (see Fig. 6), further confirming that the linear model is reasonable. In three instances, one following the exclusion of bone marrow in combination with the use of PDF and two following the exclusion of bone marrow in combination with the use of LBV, negative correlation coefficients were obtained, likely indicating the presence of artifacts.

### Comparing results in the medial and lateral condyles

Over the lateral condyle, the results obtained using V-SHARP and LBV corresponded to those seen over the medial condyle. Using PDF, however, no difference was seen between masking alternatives in this compartment. Further, a negative bias was seen between the PDF and V-SHARP techniques when no tissue was excluded; see Table 1.

## Discussion

In this work, evaluation of in vivo data was used to study the effects of excluding fatty tissue from the QSM reconstruction process in knee imaging. Several interesting findings were made: First, our results in knee-healthy subjects suggest that masking fatty tissue may decrease QSM contrast between cartilage layers, and that the used masking technique has a larger impact on results than the choice of background field removal technique. Thus, masking of fatty tissue might decrease or obscure the apparent differences between healthy and OA-cohorts reported in the previous studies [7, 12]. Second, although shading artifacts were visually detectable in the overall image only when fatty tissue was included in reconstruction, masking resulted in an increased occurrence of artifacts in the region of interest, likely affecting the QSM contrast between cartilage layers. Third, the results partly differed between the lateral and medial compartments, indicating that the conclusions may depend on geometry. Finally, if masking is necessary, e.g., if data for CSEI are not available, LBV may be advantageous compared to PDF and V-SHARP.

We are not aware of any previous comparisons between masking alternatives, but the susceptibility values presented here correspond quite well with those presented in the literature. In earlier studies, a difference in susceptibility values between the deep and the superficial zone in knee-healthy subjects of approximately 0.15 ppm has most often been reported [6–8]. In one study, Zhang et al. presented larger susceptibility differences of approximately 0.7 ppm for healthy participants [9]. For comparison, the range of susceptibility values obtained in this study was around 0.25 ppm, with the smallest being around 0.05 ppm and the largest around 0.6 ppm.

Due to the limited scope of this work, several areas remain for future studies, such as evaluation of a numerical measure of the QSM effect size in OA, other CSEI and QSM reconstruction algorithms in the knee, and the effect of imaging resolution. Below, we briefly discuss these issues.

A numerical measure of QSM contrast between layers would be valuable for use in future research studies, as this contrast has been reported to be affected by degenerative OA processes [7, 12]. So far, a few studies have used the standard deviation of susceptibility values in different cartilage compartments for between-individual comparisons [7, 10]. However, as the presence of noise and image artifacts would bias the estimated standard deviation, this measure was not suitable for this study. Instead, the slope of the increase in susceptibility with distance from the bone surface was used as a measure of the presumed effect of collagen orientation. Although this model is a simplification of the complex cartilage structure, the assumption of a linear increase of susceptibility values appears to be applicable, judging by the generally high correlation coefficients obtained. However, the model may not generally hold. Especially going toward higher resolution, a more complex model may be called for. In some cases, low, or even negative, correlation coefficients were detected. As these were not seen across all tested masking methods, we believe that they may be caused by artifacts rather than indicating that the linear model was unsuitable to describe the contrast between cartilage layers in our data. Note that the estimation of the correlation coefficient may be unreliable in cases of very few data points. Thus, care was taken to ensure that a minimum of three voxels were available for analysis in all cases.

The inclusion of fatty tissue in QSM requires CSEI prior to the reconstruction steps, as uncorrected phase data would be a source of severe artifacts stemming from fatty tissues. CSEI has mainly been implemented and validated for the separation of fat and water, whereas less attention has been put on the accuracy of the $\Delta {B}_{tot}$ estimation and its impact on QSM reconstruction. However, an accurate fat–water shift [16, 36], as well as correction from several sources of phase errors may affect the estimated $\Delta {B}_{tot}$ and thus cause artifacts in the resulting susceptibility maps [37]. Here, correction was made for phase differences between bipolar read-out gradients and a constant phase shift between the time-interleaved sequences using methods similar to those previously described [30, 31, 38], but no full correction was made for concomitant phase errors [37, 39]. If these issues affected the comparison between masking alternatives, they likely would have been beneficial for the case where fatty tissue was excluded. The impact of phase errors for QSM reconstruction are important to evaluate in future studies. A further disadvantage of the CSEI approach is the occurrence of fat/water swaps in the mDixon B0 map. Several approaches of avoiding this issue have been suggested which may be implemented for QSM purposes in future work [40].

In this work, we wanted to evaluate strategies to address fatty tissue already used in the previous studies in knee cartilage. We chose QSM reconstruction techniques based on overall popularity in the literature, and if the method allowed for no or small erosion of the mask [17]. However, several other algorithms have been suggested for body applications which may be of interest also for knee imaging. Some of these techniques both account for and utilize the presence of fatty tissue for regularization and internal reference for quantification [18–20], as well as possibly reducing shading artifacts [41]. Compared to masking, these methods would also avoid the need for erosion which may be especially challenging in the thin cartilage tissue. As we were interested specifically in the effects of fatty tissue masking, these were not evaluated here but are of interest in future work in knee QSM.

The imaging data used in this work were acquired as part of a larger study, and data acquisition could thus not be optimized solely for QSM purposes. E.g., anisotropic voxels were used as opposed to what is generally recommended [17]. Although this may affect the quality of the resulting QSM maps, it likely does not affect the comparison between masking alternatives. The impact on quantification accuracy from resolution and anisotropic voxels should be evaluated in future work.

We would like to highlight some limitations of this study. First, only knee-healthy subjects were included in this study. This choice was made as we wanted to isolate the effect of masking and therefore chose to investigate a homogenous group where we expected a contrast between cartilage layers. The ability of the investigated techniques to detect signs of OA could not be compared, nor have the results been compared to any independent measure of cartilage quality.

Second, the analysis of susceptibility values was performed using relatively small ROIs. However, we believe that this approach is suitable for OA research, where mainly the load-bearing cartilage is of interest, and, as it corresponds well with the method chosen in the other studies [6–9]. Furthermore, QSM contrast was expected to reflect the cartilage layer structure most reliably in this region, as the cartilage layers here extend orthogonally to the magnetic field of the MRI scanner. In the more anterior and posterior parts of the cartilage, as well as in the patellar cartilage, the angle to the B0 direction will alter the contrast, making it difficult to predict the expected profile over the cartilage depth. For this reason, these areas were not further evaluated here. An angular dependence of the results has also been demonstrated for other quantitative techniques in cartilage, such as mapping of T2 and T2* [42].

In conclusion, excluding fatty tissue may reduce the QSM contrast between cartilage layers and increase the incidence of artifacts in the assessment of cartilage quality of the knee. The results may, however, be dependent on geometry and the choice of reconstruction technique, and presuppose that CSEI is used to correct for phase contributions from chemical shift.

## Data Availability

Extracted measurement data (slopes and correlations) may be obtained on reasonable request. MR images are not available for public sharing due to regulations for sensitive personal data.
